# Bullets Extracted from Omentum

**Published:** 1916-10

**Authors:** 


**Bullets Extracted From Omentum.** Andre Chalier, Le Prog. Med., July 5, reports the case of an encysted ball, removed after 18 months and refers to similar cases reported by Walther, Couillod and Quenu.

